# Advancements in Nanoparticle Deposition Techniques for Diverse Substrates: A Review

**DOI:** 10.3390/nano13182586

**Published:** 2023-09-19

**Authors:** Daniel Escorcia-Díaz, Sebastián García-Mora, Leidy Rendón-Castrillón, Margarita Ramírez-Carmona, Carlos Ocampo-López

**Affiliations:** 1 Nanotechnology Engineering Program, Centro de Estudios y de Investigación en Biotecnología (CIBIOT), Chemical Engineering Faculty, Universidad Pontificia Bolivariana, Medellín 050031, Colombia; daniel.escorcia@upb.edu.co (D.E.-D.); sebastian.garciamo@upb.edu.co (S.G.-M.); 2 Chemical Engineering Program, Centro de Estudios y de Investigación en Biotecnología (CIBIOT), Chemical Engineering Faculty, Universidad Pontificia Bolivariana, Medellín 050031, Colombia; leidy.rendon@upb.edu.co (L.R.-C.); margarita.ramirez@upb.edu.co (M.R.-C.)

**Keywords:** nanomaterials, nanoparticle deposition, ceramic substrates, polymeric substrates, metallic substrates, sustainability, deposition purities

## Abstract

Nanoparticle deposition on various substrates has gained significant attention due to the potential applications of nanoparticles in various fields. This review paper comprehensively analyzes different nanoparticle deposition techniques on ceramic, polymeric, and metallic substrates. The deposition techniques covered include electron gun evaporation, physical vapor deposition, plasma enriched chemical vapor deposition (PECVD), electrochemical deposition, chemical vapor deposition, electrophoretic deposition, laser metal deposition, and atomic layer deposition (ALD), thermophoretic deposition, supercritical deposition, spin coating, and dip coating. Additionally, the sustainability aspects of these deposition techniques are discussed, along with their potential applications in anti-icing, antibacterial power, and filtration systems. Finally, the review explores the importance of deposition purities in achieving optimal nanomaterial performance. This comprehensive review aims to provide valuable insights into state-of-the-art techniques and applications in the field of nanomaterial deposition.

## 1. Introduction

The rapid development of nanotechnology has revolutionized research in applied sciences, including biomedical, cosmetic, infrastructure, electronics, and food packaging [1]. The production and use of nanomaterials are growing in various industrial processes [2,3,4]. The global market for these materials was valued at 16.3 billion dollars in 2021 [5], and it is projected to reach between 45 billion [6,7] and 62.8 billion dollars in 2031–2032 [5].

Nanomaterials are generally selected based on their desired applications. They can be synthesized through chemical, physical, or biological methods. Chemical methods involve co-precipitation, chemical reduction of metal salts, electrochemical processes (electrolysis), microemulsion, pyrolysis, photochemical reactions (irradiation), sonochemistry, sol–gel, and solvothermal techniques. Physical methods include arc discharge, electron beam lithography, ion implantation, inert gas condensation, mechanical crushing, grinding, and spray pyrolysis. Biological methods usually rely on biosynthesis approaches using plant extracts, bacteria, fungi, algae, enzymes, and biomolecules [8]. The choice of a specific method often dictates the properties of the particles and the resulting characteristics. Nanomaterials can be synthesized using a bottom-up or top-down approach [9].

Nanomaterials are generally classified based on their production into carbon-based materials, metals, dendrimers, and compounds [10]. Carbon-based nanomaterials typically contain carbon and exist in morphologies such as hollow tubes, ellipsoids, or spheres.

On the other hand, inorganic nanoparticles are produced from carbon-free materials and are classified into two major categories: metals and metal oxide nanoparticles. In most cases, nanoparticles are produced from metals such as platinum (Pt), silver (Ag), gold (Au), cadmium (Cd), cobalt (Co), iron (Fe), copper (Cu), and zinc (Zn) [9,11].

Metal-based nanomaterials are valuable and have a wide range of applications in various industries, including electronics, medical, dental, textiles, coatings, food packaging, and wastewater treatment [1,6]. Other emerging potential uses include dispersions and coatings, consolidated materials, biomedicine, and nanodevices [5], and in recent years, the development of nanomaterial applications in consumer products, such as UV filters in sunscreen, odor-resistant textiles, tumor therapies [12], lithium-ion batteries for electric vehicles, and solar panels [13].

Water treatment applications will likely increase the global demand for nanomaterials [6] and the growing demand for electronic products, mainly due to their superparamagnetic properties [14]. With the increasing application of nanomaterials, the industry is also growing, with an estimated direct employment of 300,000 to 400,000 in Europe. These materials are dominant primarily in tire manufacturing as a polymer filler, in toothpaste, or as an anticoagulant in powdered foods [13]. These applications stem from their physicochemical properties and their small size, which result in a high surface area-to-volume ratio, increased reactivity, and ideal optical properties [1], offering a larger surface area compared to the same mass of bulk materials, making them more chemically reactive [15].

Reports by Harish et al. show that nanomaterials exhibit various functionalities such as dimensionality (0–3D), morphology (low and high aspect ratio), porosity (macro-, nano-, mesoporous), composition (carbon, inorganic, organic, and hybrid), origin (natural, incidental, designed, bioinspired), phase (monophasic, multiphasic), and dispersion state (dispersed or aggregated) [16].

Studies by Chaudhary demonstrate that adding nanomaterials modifies the fundamental properties of materials, such as flexibility, durability, flame resistance, barrier properties, and recycling properties [17]. Additionally, material properties can be modified through deposition or doping [18]. This fact can lead to performance improvements, such as the deposition of an anti-reflective coating on the front surface of solar cells, reducing reflection losses by 8% [19], increasing the conductivity of semiconductors [20], improving the barrier properties of bio-based packaging materials [17], enhancing the photocatalytic activity of TiO_2_ [1], and strengthening materials [21]. Moreover, as nanoparticles are added to a common material, they refine the grain to some extent, forming an intragranular or intergranular structure, thus improving the grain boundary and promoting the mechanical properties of the materials. For example, adding 3% by weight of nano-SiO_2_ to concrete can improve its compressive strength, flexural strength, and tensile strength by splitting. Adding 3% nano-empty fruit bunch fibers to kenaf epoxy composites can significantly enhance their tensile strength, elongation at break, and impact resistance [22].

Deposition of sacrificial and structural material on the substrate is the first step in surface micromachining and can be achieved through chemical processes such as chemical vapor deposition (CVD), which is commonly used for nanomaterial manufacturing, electrodeposition, vapor phase epitaxy (VPE), and thermal oxidation, or through physical processes such as physical vapor deposition (PVD) and casting [23].

Vapor deposition methods enable a higher-purity surface coating without organometallic compounds. Currently, CVD is the most promising technology for the functionalization of nanoparticles on an industrially relevant scale. This method has multiple variations, including thermally activated, plasma-enhanced, photo-initiated, and oxidative CVD, to name just a few [24].

This review offers an in-depth analysis of the advancements in nanoparticle deposition techniques for diverse substrates, encompassing ceramic, polymeric, and metallic matrices. Additionally, it emphasizes the importance of sustainability in nanomaterial deposition and highlights potential applications in anti-icing, antibacterial power, and filtration systems. Moreover, it underscores the significance of deposition purities in attaining the optimal performance of nanomaterials. By providing a comprehensive overview of these topics, this review aims to contribute to advancing nanoparticle deposition techniques and inspire further research and innovation in this field.

## 2. Deposition of Nanoparticles on Ceramic Substrates

The deposition of nanoparticles on different substrates has garnered attention due to the changes it generates in material properties. The functionalization of ceramic substrates has enabled new applications in optical and medical fields, among others. These substrates exhibit different mechanical, optical, electrical, catalytic, and magnetic properties depending on the type of nanoparticle used for functionalization. As a result, new nanoparticle deposition techniques on these substrates have been developed, among which [25,26,27,28,29] stand out.

### 2.1. Deposition by Electron Gun Evaporation

Electron beam deposition is used to grow thin films of metals with very high melting points or highly pure metals. The precursor is evaporated with gas and then deposited onto the substrate as a film. This method has gained popularity due to its rapid deposition and ability to generate different metal or non-metal film types [30].

In the study conducted by Somayeh Jalilpour et al. [25], tantalum oxide nanolayers were prepared on a glass substrate (1 × 20 × 20) mm^3^ using an ETS160 system at a pressure of 3 × 10^−7^ Torr. The layers were obtained under a high vacuum using the electron gun evaporation method (Edwards E19A3). The purity of tantalum oxide was 99.9%, and the deposition rate was 0.2 Å/s. The substrate temperature was kept constant at 300 K. Before the deposition process, the substrates were cleaned with deionized water, acetone, and ethanol for 15 min each using an ultrasonic method. The thickness of the layers (30, 60, 90, and 120 nm) was measured using a quartz crystal technique (Sigma Instrument, SQM–160, Fort Collins, CO, USA) from the deposition angle of the layers, which was vertically oriented.

The study on the coatings’ crystalline nature and morphological evolution was characterized using X-ray diffraction (XRD) and field emission scanning electron microscopy (FESEM), respectively. Surface morphology and roughness were obtained through analysis using an atomic force microscope (AFM) [25].

### 2.2. Physical Vapor Deposition (PVD)

**Figure 1:**
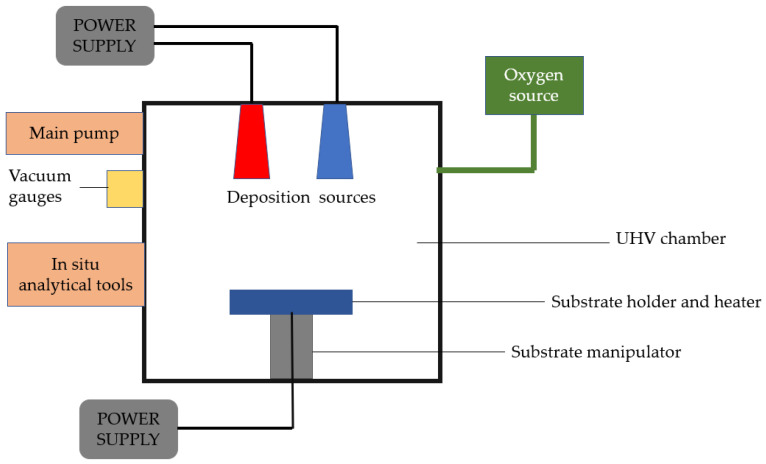
Schematic of a fundamental physical vapor deposition system, reinterpreted from [32].

Physical vapor deposition is a vaporization coating technique that involves the transfer of material at the atomic level under vacuum conditions. This technique can be applied to metals, ceramics, and semiconductors. The process is similar to chemical vapor deposition (CVD), except that physical vapor deposition uses a solid precursor instead of a gas-phase precursor in CVD. The process consists of four stages: the evaporation of the material to be deposited or the precursor using a high-energy source such as an electron beam, the transport of vapor to the substrate, the reaction between the substrate atoms and the reactive gas, and finally, the deposition of the coating onto the substrate surface [31] (See Figure 1).

In the study conducted by Sanath Kumar Honnali et al. [33], thermoelements were fabricated using glass as the substrate. The substrate underwent a wet chemical cleaning process followed by steam degreasing. Subsequently, the chamber was loaded with O_2_ to perform a 15-min plasma cleaning, varying the pressure from 8 × 10^−2^ mbar to 1 × 10^−1^ mbar. This plasma cleaning increases the Si–O functional groups, thus enhancing the adhesion of thin films on the substrate. Then, ZnO was deposited in the cathodic sputtering with pure zinc as the target at a substrate-reactive distance of 5 cm. The chamber was evacuated to a base pressure of 2 × 10^−5^ mbar to minimize residual background impurities. Several deposition cycles were performed at different oxygen partial pressures. The pressure ranged from 9 × 10^−4^ mbar to 3 × 10^−3^ mbar, maintaining a total working pressure (O_2_ + Ar) of 8 × 10^−3^ mbar for each deposition cycle. A power of 16 W with a deposition rate of 10 nm/min and a deposition time of 10 min resulted in a film thickness of 100 ± 10 nm.

### 2.3. Plasma-Enhanced Chemical Vapor Deposition (PECVD)

**Figure 2:**
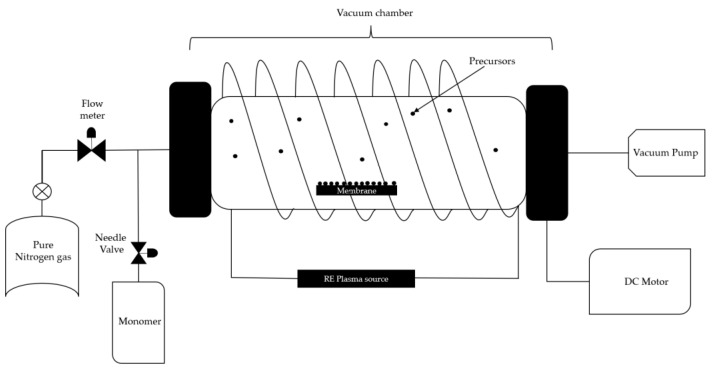
Chemical vapor deposition with enriched plasma. Reinterpreted from [35].

Plasma-enhanced chemical vapor deposition is known as a fast and clean technique for surface modification, where plasma is implemented to achieve more localized and controlled deposition on the substrate. This technique is commonly used on silicon templates to coat them at low temperatures with the desired material and can be applied also to ceramics and polymers. Figure 2 shows a schematic of the plasma-enhanced chemical vapor deposition, where, from the equipment perspective, an inert gas flow is used to prevent secondary reactions, along with a vacuum system and a magnetic field. From the material perspective, the precursor is to be deposited, followed by the target substrate [34].

In the study conducted by Noresah et al. [35], they report the functionalization of an ultrafiltration membrane by depositing two monomers onto the membrane surface under vacuum conditions. A cylindrical quartz vacuum chamber measuring 30 cm in length and 6 cm in diameter was used as the reactor. The UF membrane sample (Dimensions: 6 cm × 15 cm) was placed on flat silicon wafers before being transferred to the chamber. A copper coil antenna connected to a 13.56 MHz radiofrequency plasma generator was positioned through the quartz window of the chamber. Firstly, a vacuum condition was created inside the chamber by turning on the vacuum pump to remove the air from the reactor. The monomer 2-hydroxyethyl methacrylate was vaporized in a stainless steel flask before being delivered to the vacuum chamber by controlling the fine metering valve. A back cooling plate was installed as a substrate support to maintain the membrane temperature. The chamber’s desired operating pressure (<100 mtorr) was maintained using a proportional integral derivative-controlled butterfly valve connected to a capacitance manometer. The optimized flow rate and power of acrylic acid plasma (0.75 sccm; 40 W) and the deposition of 2-hydroxyethyl methacrylate (0.4 sccm; 50 W) were used throughout the modification process.

There are additional deposition methods besides those mentioned previously. Table 1 summarizes ceramic substrates’ most relevant nanoparticle deposition methods, including the Tollen reaction, electrochemical deposition, electron beam gun evaporation, spray deposition, and electrolytic deposition. The structural properties of the functionalized substrate are reported, along with the different techniques, their respective variables, and their applications in the industry.

Some methods stand out for their low cost, such as electrochemical deposition or spray deposition. Electron beam gun evaporation allows the production of nanolayers, which are widely used for their nonlinear optical properties that change depending on the thickness of the obtained film. This method, in turn, modifies the material’s dielectric properties since increasing the film’s thickness increases the material’s bandgap [25]. The electrochemical deposition also imparts materials with nonlinear optical and photoelectronic properties, which depend on the morphology of the obtained nanowires. These properties enable applications in sensors, solar cells, and thin film transistors; this technique is highlighted as a low-cost alternative [27].

## 3. Deposition of Nanomaterials on Polymeric Substrates

The functionalization of polymeric substrates, similar to ceramic substrates, has allowed for new applications of these materials in various areas, including medicine, photonics, and sensors. These substrates exhibit different thermal, mechanical, optical, and catalytic properties depending on the nanomaterial used for functionalization. This fact has led to the development of new nanomaterial deposition techniques on this type of substrate [36,37,38,39,40,41,42,43,44,45,46]. The most prominent techniques are presented below.

### 3.1. Simultaneous and Consecutive Electrochemical Deposition

**Figure 3:**
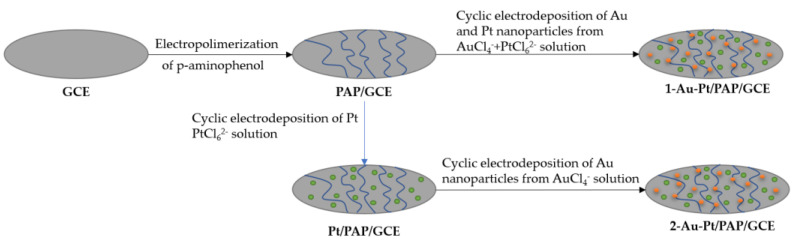
Procedure to design the Au Pt/PAP/GCE. Reinterpreted from [46].

Nanoparticle electrochemical deposition can be performed through two different pathways, as shown in Figure 3; they are known as simultaneous electrochemical deposition and consecutive electrochemical deposition [46]. This technique is applicable also to metal and to ceramic matrices.

#### 3.1.1. Simultaneous

In this method, nanoparticles of two different types are simultaneously deposited, as the name implies. As reported by Zekerya Dursun et al. [46], electrodes are doped with gold and silver nanoparticles by immersing two electrodes in a solution of chloroauric acid, which serves as the precursor for gold nanoparticles, and potassium hexachloroplatinate, which serves as the precursor for silver nanoparticles. A potential of 0.8 V to −1.1 V is then applied for ten cycles to produce the doping.

#### 3.1.2. Consecutive

In this technique, unlike simultaneous deposition, the process is performed in stages, doping first with one material and then with the other. Taking the case of the study by Zekerya Dursun et al. [46], for this method, immersion in a solution of potassium hexachloroplatinate is performed with a potential of 0.8 V to −1.1 V, followed by immersion in a solution of chloroauric acid with a potential of 0.1 V to −0.9 V, each for ten cycles.

### 3.2. Chemical Vapor Deposition (CVD)

Chemical vapor deposition follows the same principle as Enriched Chemical Vapor Deposition, the difference being that PECVD introduces precursors with a plasma gas capable of ionizing the precursors. In contrast, CVD introduces chemical precursors into the reaction chamber that thermally decompose to form solid films without plasma [34].

**Figure 4:**
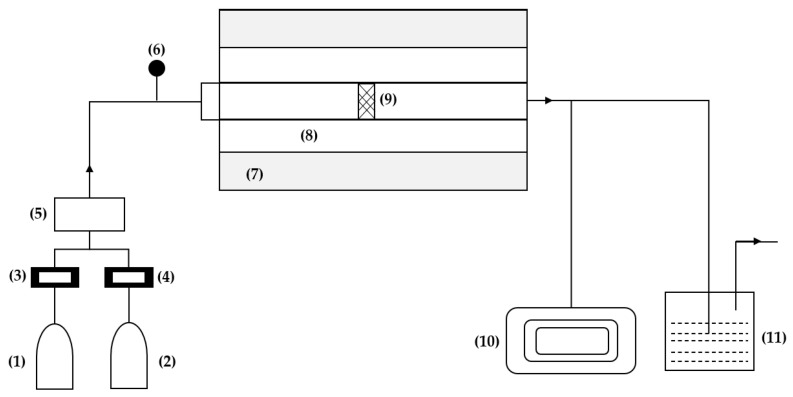
Experimental deposition system: (1) argon, (2) propylene, (3) flow meter, (4) manometer, (5) mixing cylinder, (6) manometer, (7) furnace, (8) graphite reaction chamber, (9) carbon layer, (10) vacuum pump, (11) off-gas treatment device. Reinterpreted from [37].

The experimental system reported in the study by Hui Kun et al. [39] for this technique consists of six parts, as shown in Figure 4: a gas device, a high-temperature furnace, a graphite reaction chamber, a carbon layer sample, a vacuum pump, and a tail gas treatment device. The gas system is divided into two parts, one for argon and the other for propylene, each equipped with a flow meter. The graphite reaction chamber comprises a front graphite channel, a front reaction chamber, a rear reaction chamber, and a rear graphite channel. This design ensures that the reaction gas passes through the porous structure of the carbon layer sample completely.

In the study by Hui Kun et al. [39], a temperature of 1600 °C is reported inside the furnace, which is maintained stable for 30 min, and the temperature in the graphite reaction chamber is approximately 50 °C lower than that of the furnace. A high-temperature chemical reaction will cause mass loss in the carbon layer, so comparative experiments are performed to reduce the error.

The process is divided into four stages. First, the furnace is evacuated using the vacuum pump, then argon is introduced until the pressure exceeds atmospheric pressure. Once reached, the outlet valve is opened, and the furnace is heated to the reaction temperature with the reaction gas. At this point, the argon valve is closed. At the end of the reaction, the reaction gas valve is closed, and the argon valve is opened again [39].

### 3.3. Electrophoretic Deposition

Electrophoretic deposition is a widely used technique that promises cost-effective deposition of nanoparticles with relevant applications, such as obtaining antibacterial properties and improving the biocompatibility of a ceramic compound. It is used to uniformly coat an electrode immersed in a stable suspension. The cathode and anode are subjected to an electric field, inducing the movement of precursor particles to the substrate [47]. The electrophoretic deposition can be applied to ceramic, metal, and polymer substrates.

**Figure 5:**
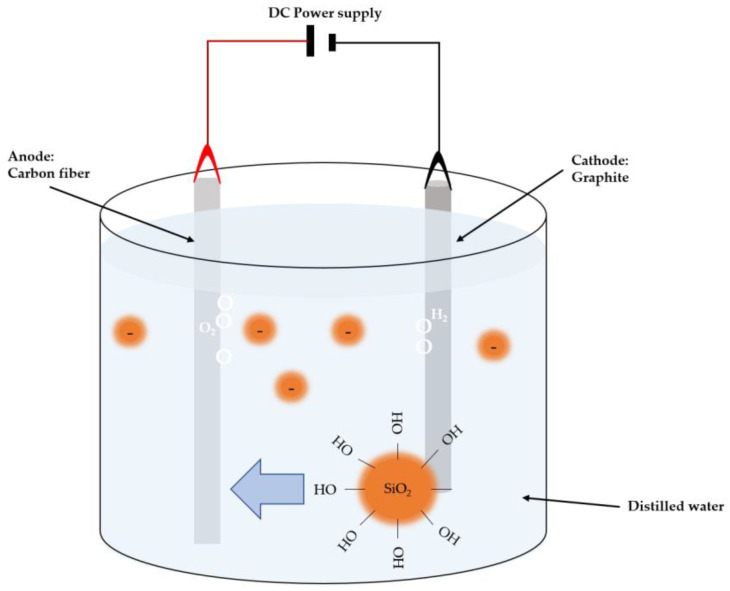
Schematic illustration of EPD configuration for CF surface modification.

Figure 5 illustrates the setup used by Huanyu Li et al. [40] for electrophoretic deposition, where doping of nano-silica onto carbon fibers is performed. A carbon fiber anode is immersed in distilled water containing nano-silica colloids, and a graphite cylinder is used as the counter electrode. In this technique, voltage and time parameters can be varied, typically ranging from 0 V to 3 V throughout 0 to 15 min, depending on the desired doping, followed by drying of the specimens.

There are more deposition methods in addition to those previously mentioned. Table 2 reports different techniques with their respective variables and applications in the industry.

The application techniques employed showcase the strategic deployment of nanomaterial deposition to achieve tailored functional enhancements. Notably, Chemical Vapor Deposition (CVD) demonstrates the potential to create nanoporous structures in polymer composites, substantially elevating mechanical properties and resistance to erosion [39]. On the other hand, Electrophoretic Deposition (EPD) stands out for its adeptness in enhancing bond properties between nano-silica and carbon fiber surfaces [40]. It stands out for its low cost, simplicity of operation, and the possibility of applying different coating materials. It is also used to improve the performance of interfacial bonds between a substrate and a precursor, thus improving the mechanical properties.

In situ deposition techniques, including creating antimicrobial properties through nano-Cu_2_O on chitosan nanofibrous scaffolds, exemplify the versatility of these methodologies. Additionally, the ingenious application of the sol–gel method to deposit NiO nanoparticles on cotton fibers imparts notable photocatalytic activation properties, opening avenues for environmental conservation [41,42]. Pulsed Laser Deposition (PLD) emerges as a promising contributor to wound healing, with the deposition of silver nanoparticles onto specific scaffolds showcasing enhanced antibacterial activity [43].

The strategic selection of nanomaterials and matrices further underlines the potency of these techniques, with examples such as nano-silica on carbon fiber offering improved mechanical properties, nano-Cu_2_O on chitosan nanofibrous scaffolds displaying potential for wound healing, and NiO nanoparticles on cotton contributing to environmental conservation.

A notable method involves depositing photocatalytic micron-thick TiO_2_ coatings using a microblast technique on titanium metal and FTO-coated glass substrates. This technique demonstrates scalability and cost-effectiveness, showing potential for practical applications. In enhancing adhesion and inter-particle connectivity of TiO_2_ coatings, both furnace and microwave plasma treatments have been explored. Plasma-treated TiO_2_ coatings exhibit increased particle packing density, resulting in substantially improved photocurrent density measurements compared to their as-deposited counterparts, thus elevating the application possibilities of this technology.

In a separate study, {[PMo_12_O_40_]^3−^/PAMAM}_n_ multilayer films are synthesized through an LBL electrostatic assembly technique. These films, marked by uniformity and homogeneity, provide a matrix for the in situ electro-deposition of Pt micro–nano clusters. The resulting Pt micro–nano clusters with a unique flower-like structure become immobilized on the surface of the {[PMo_12_O_40_]^3−^/PAMAM}_n_ multilayer films. Electro-deposition conditions, such as deposition potential and time, influence the morphology of Pt micro-nano clusters. The resultant Pt-clusters–{PMo12/PAMAM}3 composite films exhibit robust electrocatalytic activities, particularly in methanol oxidation and improved CO tolerance [45].

Furthermore, the direct electrochemical oxidation of sodium borohydride is investigated through Au–Pt-nanoparticles-decorated poly(p-aminophenol) (PAP) films. These bimetallic surfaces, prepared using simultaneous and successive electro-deposition procedures, serve as catalysts for borohydride electrochemical oxidation. Simultaneous electro-deposition of Au–Pt onto the polymer structure enhances oxidation potential and current density. Extensive characterization, including AFM, SEM, XPS, XRD, and CV, sheds light on these modified surfaces’ structural and morphological properties. The outcomes underscore the potential of these bimetallic catalysts for advancing electrochemical applications, offering insights into their catalytic activities and electron exchange numbers [46].

## 4. Deposition of Nanomaterials on Metallic Substrates

The functionalization of metallic substrates has allowed for new applications of these materials in optical, medical, environmental, and other fields. These substrates exhibit different mechanical, optical, electrical, catalytic, and magnetic properties depending on the type of nanoparticle used for functionalization. As a result, new nanoparticle deposition techniques have been developed for this type of substrate [10,26,27,28,29,30,31,32,33,34,35,36,37,38,39,40,41,42,43,44,45,46,47,48,49,50,51,52,53,54,55,56,57,58,59,60,61,62,63,64,65,66,67,68,69]. The most prominent ones are presented below.

### 4.1. Laser Metal Deposition

**Figure 6:**
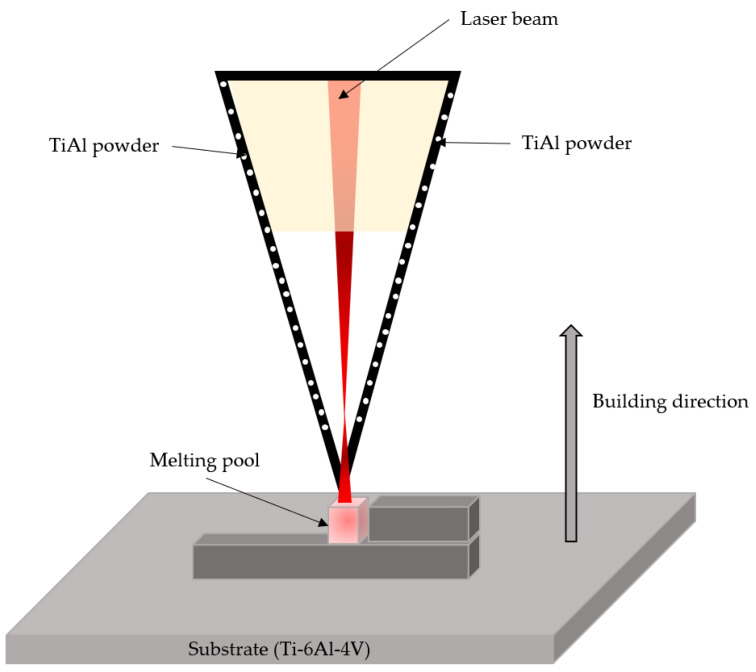
Schematic illustration of the LMD process. Adapted from [49].

This technique is mainly based on a fiber laser with a power of ~6000 W that irradiates the substrate while directing the powder of the desired doping material, including metal, ceramic and polymer substrates. Figure 6 shows the laser metal deposition (LMD) process diagram. This technique provides high precision and a rapid deposition rate [48].

The study by Qi Zhang et al. [49] reports doping a Ti–6Al substrate with a mixture of TiB_2_/Ti64 powders. The system consisted of a diode laser, a rotating powder feeder, and an industrial robot. The laser power used was 1300 W, with a beam diameter of 3.5 mm and a scanning speed of 10 mm/s. The scanning overlap distance between adjacent paths was 1.92 mm, corresponding to a 40% overlap ratio. The powder feed rate and corresponding layer height were 6.5 g/min and 0.7 mm, respectively. Additionally, the temperature of the Ti64 substrate was 480 °C. All parameters were optimized to prevent cracking of the titanium matrix.

### 4.2. Electrochemical Deposition

Electrochemical deposition is similar to electrophoretic deposition as it occurs in an electrochemical cell. It is carried out using different configurations with electrodes submerged in an electrolyte mixed with the precursor, which is then subjected to a voltage to induce doping. In the study by C. Augello et al. [50], the substrate was immersed in a solution of monomers acting as precursors. When the voltage was applied, the monomers underwent chemical polymerization on the substrate. This method offers advantages such as improving the interfacial bond between the coating material and the substrate.

### 4.3. Atomic Layer Deposition (ALD)

ALD is a particular variant of the CVD technique. In ALD, gas-phase reactants are also introduced into a reaction chamber to form the desired material through surface chemical reactions. The difference is that, in ALD, the precursors are pulsed alternately, one at a time, with an intermediate purge of inert gas [51]. This technique is based on sequential and self-limiting surface reactions. Most ALD processes involve binary reaction sequences where two surface reactions occur to form the layer. This method offers advantages such as precise control over film thickness and can be applied to metal and ceramic substrates [52].

In the study by Sheng-Hao et al. [53], the substrate was deposited in a commercial ALD system with the precursor and H_2_O at a chamber temperature of 200 °C. The precursor container was heated to 70 °C, and the H_2_O container was kept at room temperature during the ALD processing. A cycle consisted of six steps: precursor 1/dosing time, soak time, evacuation time; precursor 2/dosing time, soak time. During ALD processing, there is a constant flow of carrier gas.

In addition to the methods mentioned above, there are other deposition techniques. Table 3 presents different techniques and their respective variables and applications in the industry. These techniques include electrophoretic deposition, chemical vapor deposition, electrochemical deposition, atomic layer deposition, and laser metal deposition, among others.

From Table 3, valuable insights were obtained regarding applying various nanomaterial deposition techniques and their impact on different matrices. Electrostatic layer-by-layer deposition utilizing diamond nanoparticles (SiO_2_, TiO_2_) on a Si substrate has shown promise in improving mechanical properties. Plasma-assisted filament evaporation with silver nanoparticles on an indium tin oxide (ITO) matrix demonstrates the ability to enhance optical and electrical properties. Direct laser deposition employing nano-TiB whiskers on Ti–6Al–4 V exhibits improved mechanical properties. Electrochemical deposition of Si nanospheres on Au and graphene-coated Cu showcases the potential for high reproducibility.

Furthermore, chemical vapor deposition of carbon nano-onions on Fe/Fe_3_C matrices offers corrosion protection, while atomic layer deposition of nano Sn–O_2_ on titanium demonstrates biocompatibility. Laser metal deposition with nano-SiCp on AlSi10Mg alloy influenced the microstructure, decreasing SiCp size and intensifying the reaction between SiC and the aluminum matrix. Other notable findings include electrophoretic deposition of chitosan-reinforced Baghdadite ceramic nanoparticles on stainless steel 316 L to improve biological and physical characteristics, electrophoretic deposition of polyaniline (PANI) nanofibers on copper for corrosion protection, and in situ deposition of Pt nanoparticles on nanopore stainless steel for high active hydrogen evolution reaction. These findings contribute to a comprehensive understanding of nanomaterial deposition techniques and their potential applications, facilitating the development of advanced materials with tailored properties in nanotechnology.

The CVD stands out as an economical and effective alternative for the deposition of nanomaterials. It allows for the modification of different materials by providing nanostructures on their surface. For example, nitride semiconductors like gallium nitrides and aluminum nitrides can be produced using CVD, forming nanostructures known as quantum confinement points. These nanostructures allow the materials to be utilized in a wide range of optical and microelectronic devices [87].

## 5. Other Techniques for Nanomaterials Deposition

### 5.1. Thermophoretic Deposition

Thermophoretic deposition brings forth advantages that enhance its applicability and versatility. One key advantage is the capability for selective deposition, driven by the distinct thermophoretic behaviors exhibited by nanoparticles of differing sizes, compositions, and surface chemistries. This fact allows researchers to intentionally deposit specific nanoparticle types onto substrates, tailoring the composition for targeted functionalities. Moreover, the non-contact nature of thermophoretic deposition proves advantageous, as it sidesteps the need for physical contact between nanoparticles and the substrate. This characteristic becomes particularly valuable when dealing with delicate or sensitive substrates that direct contact may adversely affect.

Thermophoresis facilitates the adhesion of small particles onto a chilled surface. In their study, Alam et al. [94] investigated the effects of thermophoresis and turbulent suction on a 2D (two-dimensional) steady magnetofluid flow over an inclined semi-infinite plate. In a study by Rahman [95], the interplay between thermophoretic particle deposition and magnetic effects was showcased within a nanofluid flowing through a rotating system. The research revealed that the particle deposition rate driven by thermophoresis is substantially shaped by factors encompassing thermal diffusion, slip mechanisms, magnetic fields, diffusion-thermo, and radiation. Additionally, recent investigations [96,97] have further elucidated the ramifications of thermophoretic particle deposition across a spectrum of distinct scenarios.

### 5.2. Supercritical Deposition

Depositing metals and metal oxides onto surfaces using supercritical fluids (SCFs), particularly supercritical carbon dioxide (scCO2), has gained significant attention due to SCFs’ appealing properties. The advantages of utilizing scCO2 as a deposition medium include adjustable solvent power via pressure and temperature changes, absence of liquid waste, no solvent residue on substrates, rapid mass transfer rates facilitating swift deposition, low surface tension, and seamless miscibility with reactive gases like hydrogen or oxygen.

A broad range of metals and metal oxide nanoparticles or films, such as Cu, Co, Ni, Mo, Pt, Pd, Ru, Ag, Au, Rh, and various oxides like HfO_2_, ZrO_2_, yttria-stabilized zirconia, ceria, titanium, tantalum, niobium, and bismuth oxide, have been successfully deposited on diverse support materials including polymers, carbon nanotubes, graphene, carbon blacks, aerogels, alumina, silica, and silicon. Among these, Pt has been extensively studied due to its high catalytic activity and wide use in fuel cells and hydrogenation. In particular, carbon-supported Pt electrocatalysts prepared through supercritical deposition exhibit exceptional efficiency in oxygen reduction and hydrogen oxidation reactions. Comprehensive reviews of supercritical deposition research have been conducted over the past decade, underscoring its significance [98]. This technique is applied to ceramic, polymer, and metal substrates.

### 5.3. Spin Coating

The spin-coating method is an effective technique for depositing nanoparticles onto various substrates. Gorji [99] found that precise control of spin-coating parameters, such as spinning speed and duration, is necessary to optimize the density and distribution of gold nanoparticles on silicon substrates. Colson [100] applied an experimental design to spin coating and found that the degree of ordering in nanoparticle monolayers is positively correlated with ramp time and negatively correlated with the first rotation speed. Weiss [101] demonstrated that spin coating can produce rough surfaces of uniform nanoparticles. At the same time, Bräuer [102] showed that spin coating can deposit thin films of magnetic transition metal complexes with preserved molecular structure.

### 5.4. Dip Coating

The nanoparticle deposition by dip coating is a viable method for creating coatings. Sinturel [103] found that the presence of PLGA nanoparticles in PVA and PVP water solutions did not modify the film deposition process by dip coating. Prevo [104] reviewed a convective assembly technique for rapid deposition of structured micro- and nanoparticle coatings, which can be applied to various colloidal systems. Chun [105] developed a nanoparticle deposition system (NPDS) for ceramic and metal coating at room temperature and low vacuum conditions. This fact provides a new coating method for ceramic and metal materials with a large surface area. Shukla [106] investigated a cold gas dynamic spray (CGDS) technique for nanopowder deposition, which produced copper and nano-WC/10% Co coatings on steel and aluminum substrates.

## 6. Overview of the Driving Forces for the Deposition and Transformation of Nanoparticles

The successful implementation of nanoparticle deposition techniques relies on a nuanced understanding of the underlying forces that drive nanoparticle deposition and subsequent transformation. These driving forces can be broadly categorized into several key mechanisms, each influencing the spatial arrangement and properties of the deposited nanoparticles.

- Surface Interaction Forces: Surface interaction forces govern the affinity of nanoparticles for a substrate’s surface. Van der Waals forces, electrostatic interactions, and capillary forces are common surface-driven mechanisms. Van der Waals forces arise from temporary fluctuations in electron distribution, leading to attractive interactions between particles and substrates. Electrostatic interactions, influenced by the charge distribution of particles and substrate, contribute to adsorption or repulsion. Due to surface tension gradients, capillary forces direct nanoparticles to specific locations on the substrate [107].
- Chemical Affinity: The chemical affinity between nanoparticles and substrates arises from the compatibility of their surface chemistry. In cases where nanoparticles and substrates possess complementary functional groups, chemical bonds can form, enhancing adhesion. Such interactions are vital in processes like plasma-enhanced chemical vapor deposition (PECVD) and atomic layer deposition (ALD), where precursor molecules chemically react on the substrate’s surface to yield conformal coatings [108,109].
- Electric Fields: Electric fields are potent tools for nanoparticle manipulation and deposition. Electrostatic deposition methods, such as electrophoretic deposition, exploit the attraction of charged nanoparticles to oppositely charged electrodes. This method allows for controlled positioning and uniform deposition of nanoparticles on substrates [110,111].
- Thermal Effects: Temperature gradients and localized heating can induce thermal motion of nanoparticles, influencing their deposition. Techniques like laser metal deposition and electron gun evaporation utilize thermal energy to vaporize or melt nanoparticles, which then condense onto the substrate surface [112,113].
- Fluid Dynamics: Fluid flow and dynamics play a significant role in spin- and dip-coating techniques. The controlled spreading and receding of liquid suspensions on substrates lead to an even distribution of nanoparticles. Fluid dynamics also underlies thermophoretic and supercritical deposition methods, where carrier gases or fluids carry nanoparticles to specific deposition sites [99,101,102,103].
- Kinetic Factors: Deposition kinetics determine the rate and extent of nanoparticle assembly. Factors like deposition time, precursor concentration, and growth rate influence the morphology and coverage of deposited layers. In techniques such as physical vapor deposition (PVD), carefully controlling these parameters is essential to achieve desired film characteristics [31,32,33].

Table 4 summarizes the driving forces for the deposition and transformation of nanoparticles for each discussed technique.

## 7. Sustainable Approaches for Nanomaterial Synthesis

The irregularities in nanomaterials’ shape, size, and chemical composition raise concerns about their adverse effects on the environment and human health. The fate, transport, and transformation of nanoparticles released into the environment are critical areas of study [114].

There are sustainable approaches for nanomaterial deposition. Varma [115] highlights using naturally occurring biodegradable materials as reducing and capping agents for nanoparticles, which can reduce toxicity. Xu [116] discusses various green methods for producing photo-active nanomaterials, such as hydrothermal methods and ultrasound sonication, which minimize the use and generation of hazardous substances during manufacturing. Singh [117] emphasizes the need for sustainable nanomaterials to overcome the challenges associated with engineered nanomaterials and discusses their applications in catalysis and corrosion control. Luque [118] advocates for more thoughtful and carefully designed methodologies that consider environmentally sound protocols for nanomaterials development, such as low-temperature ambient pressure methods and the avoidance of hazardous chemicals.

Recognizing the potential advantages and unintended risks associated with nanomaterials synthesis is crucial for their responsible development. While nanotechnology has shown promise in numerous applications, the increasing presence of nanomaterials in the environment raises environmental pollution concerns. Preliminary studies indicate that nanomaterials can affect air, water, and soil quality and disrupt the environmental system’s life cycle [114].

When synthesizing nanomaterials, a significant amount of chemical waste is generated, which poses environmental pollution concerns. Conventional methods like coagulation, filtration, and electrocoagulation have been developed to remove impurities, including chemicals and heavy metals, released into the environment to address this issue. However, these methods could be more efficient and require modifications to eliminate smaller particles effectively. Therefore, the sustainability of synthesis methods becomes crucial to minimize waste generation and enhance efficiency in nanomaterial fabrication and waste treatment processes [119].

Specific nanomaterials have been employed in water desalination applications, necessitating low-cost methods to combat the global challenge of water salinity impacting communities worldwide. Scalable solutions that can meet the global demand are imperative, leading to the utilization of non-permeable membrane solutions incorporating nanomaterials obtained through sol–gel and chemical vapor deposition (CVD) techniques. These methods have been recognized as the most scalable and cost-effective approaches for such applications [120].

Moreover, a cost-effective plasma cannon method has been employed to deposit a novel compound onto a commercial cotton surface. This method is considered low-cost as it aims to reduce energy consumption and the typically high temperatures associated with such processes. The objective is to develop an innovative, single-step method capable of depositing nanoparticles onto flexible surfaces to create electrically conductive coatings [121].

The CVD method holds promise for reducing manufacturing costs due to its applicability to large surfaces and the potential for adjusting working parameters to minimize energy consumption and improve deposition efficiency [122].

Coating-based methods, such as dip coating and spin coating, are frequently employed to modify electrodes with nanomaterials, serving as cost-effective approaches for biosensor fabrication, as they do not require complex equipment. Direct deposition methods encompass electrochemical, electrospinning, electrospray, sputtering, and chemical vapor deposition. These methods offer better control over output parameters and do not require a vacuum pump. Furthermore, the printing-based method represents a novel technique for depositing nanomaterials onto rigid and flexible substrates, enabling large-scale production of devices at a low cost (e.g., screen printing, inkjet printing) [123].

It is essential to highlight that the abovementioned methods effectively address various challenges associated with conventional approaches, including high energy requirements, complex equipment, and low efficiency. While methods like atomic layer deposition (ALD) offer precise control over deposition, the methods discussed above are more efficient and cost-effective for large-scale production [95]. Sustainability plays a vital role in these applications, as the products must be affordable without incurring high production costs.

## 8. Potential Applications in the Deposition of Nanomaterials

As shown in this study, it is evident that nanomaterial deposition on different types of substrates is often carried out to enhance a specific property (mechanical, electrical, corrosion resistance, photocatalytic, and biocompatibility, among others) of the material and adapt it to a specific application. Therefore, some applications that utilize deposition methods to improve properties directly influencing composite material behavior will be mentioned below.

### 8.1. Sensing

The development of modified electrodes has been crucial in creating a new generation of analysis and sensing devices with greater sensitivity and selectivity. Several studies mention using nanomaterials for sensing applications where a material with significant changes in response to minimal variations in a medium or stimulus is desired [124,125]. For instance, indium oxide is commonly employed as an n-type semiconductor for detecting pollutant gases. However, previous studies have shown lower selectivity and sensitivity for gases than other elements. A zinc-doped indium oxide nanowires nanocomposite was obtained through CVD to enhance sensitivity and selectivity. The doping with zinc increased electrical resistivity, which is essential for sensing applications [126].

### 8.2. Anti-Icing

Ice accumulation on brass surfaces can lead to heat transfer inefficiency, equipment degradation, and potential accidents [127]. Superhydrophobic surface technologies are employed to mitigate these effects to create anti-icing surfaces. Laser ablation is utilized to deposit silica nanoparticles, resulting in anti-icing properties. The anti-icing effect is influenced by surface structure, droplet size, and surface temperature. An increase in the apparent contact angle delays the freezing process [127]. The deposition of silica nanoparticles and treatment with a nanolaser achieved an apparent contact angle of 164.5°, leading to a delayed freezing process. However, superhydrophobic coatings are susceptible to damage during repeated ice formation and melting cycles, raising concerns regarding the durability and efficiency of anti-icing coatings [128].

### 8.3. Antibacterial Power

The issue of bacterial resistance to antibiotics poses a significant problem in human health, leading to research on alternative antibacterial strategies that do not rely on antibiotics [129,130,131]. Carbon nanotubes with vertical alignment are proposed as a nanomorphological design to destroy bacteria [129]. The antibacterial power of vertically aligned carbon nanotubes has been demonstrated, with the ability to achieve 100% inactivation of bacteria. These nanomaterials present a potential alternative to antibiotics and can be applied to self-cleaning surfaces for the prevention of microbial colonization [129].

### 8.4. Filters

Particulate matter is a critical concern when addressing air pollution. In one study, a compound consisting of lightweight and flexible carbon nanotubes is proposed for use as filters in particulate matter removal [132]. CVD with a floating catalyst is implemented to obtain carbon nanotubes for their application as particulate matter filters. The composite filter composed of carbon nanotubes exhibited an efficiency of over 90% for particles with a size of 2.5 µm. It is a lightweight and flexible filter suitable for textile applications or room filters [132].

## 9. Purity Requirements for Precursors Used in Deposition Techniques

It is crucial for the precursors in a deposition process to be as pure as possible to prevent impurities from affecting the desired properties. Table 5 presents the required purities of precursors employed in different deposition techniques. These impurities can vary depending on the sintering methods of the nanostructures. Commonly used physical reduction methods for nanoparticle synthesis may introduce traces of other metals from the reduction equipment or the same reduction process. Different types of mills, such as planetary and high-energy ball mills, are commonly used for this purpose. Similarly, chemical reduction synthesis may also contain traces depending on the reactants and washing steps performed during the synthesis [133,134].

Impurities can significantly affect the quality and properties of the resulting nanomaterials. In vertical deposition, impurities lead to uneven growth, causing thickness, density, and composition variations along the vertical axis, compromising structural integrity and functionality [25]. Plasma-enhanced deposition is affected by impurities altering plasma chemistry, leading to unintended elements in the nanomaterial. This phenomenon changes its properties, making it unsuitable for specific applications [28].

In pulsed laser deposition, impurities disrupt laser interactions, affecting ablation and nanomaterial composition. This results in reduced crystallinity, changed surface structure, and weaker mechanical properties [43]. Physical vapor deposition sees impurities contaminating the deposition chamber and film, causing changes in microstructure, electrical conductivity, and thermal properties, thus limiting potential uses [36].

Electrophoretic deposition with impure suspension leads to uneven particle coating, reduced adhesion, and mechanical stability. Electroless deposition’s catalytic reactions are hindered by impurities, causing incomplete or non-uniform coating and affecting surface properties and catalytic ability [139]. Impurities in atomic layer deposition disrupt self-limiting reactions, causing inconsistent film growth and altering nanomaterial properties [109]. Maintaining high precursor purity is essential for desired nanomaterial characteristics and performance in all cases.

## 10. Conclusions and Perspectives

In conclusion, the deposition of nanoparticles onto diverse ceramic substrates has unlocked transformative material property changes, driving innovations in optical, medical, and other fields. Notably, electron gun evaporation, physical vapor deposition (PVD), and plasma-enhanced chemical vapor deposition (PECVD) have emerged as potent techniques for controlled deposition, each offering unique advantages. The studies around this topic underscore the significance of substrate-catalyzed reactions and tailored deposition processes. These advancements expand the arsenal of nanoparticle deposition methods, with low-cost alternatives like electrochemical deposition and spray deposition gaining traction, enabling applications in sensors, solar cells, and more. This article opens avenues for developing enhanced materials and devices across industries as these techniques evolve.

Advanced nanoparticle deposition techniques have expanded the use of polymeric substrates in various fields like medicine, photonics, and sensors. Electrochemical deposition methods, including simultaneous and consecutive approaches, enhance materials by introducing multi-metal nanoparticles into electrodes. Chemical vapor deposition (CVD) demonstrates the potential to strengthen polymer composites through nanoporous structures. Electrophoretic deposition (EPD) enhances bonding, while in situ deposition methods like adding nano-Cu_2_O to chitosan nanofibrous scaffolds demonstrate versatile functional improvements. These techniques highlight the synergy between nanomaterials and matrices, with applications spanning from wound healing to photocatalysis. Innovative methods like microblast deposition and electro-deposition of Pt micro–nano clusters onto multilayer films offer cost-effective routes for practical advancements.

Functionalizing metallic substrates using advanced nanoparticle deposition techniques has revolutionized their applications across diverse sectors such as optics, medicine, and the environment. These substrates exhibit mechanical, optical, electrical, catalytic, and magnetic properties, which can be tailored through innovative deposition methods. Laser metal deposition (LMD) enables precise and rapid material incorporation, while electrochemical deposition enhances interfacial bonds. Atomic layer deposition (ALD) allows for controlled film thickness. The findings from various deposition techniques provide comprehensive insights into their potential applications, such as enhanced mechanical properties, corrosion protection, and improved biocompatibility. Notably, Chemical vapor deposition (CVD) is a versatile and cost-effective approach, offering a broad spectrum of nanostructured surface modifications. Collectively, these advancements hold the key to tailoring material properties for diverse nanotechnological applications, with CVD playing a pivotal role in expanding material functionalities.

In nanomaterial deposition, addressing environmental and health concerns is crucial, and sustainable methods are emerging as effective solutions. These approaches involve using biodegradable materials for reduction and capping alongside eco-friendly techniques. Emphasis is placed on the significance of sustainable nanomaterials to overcome challenges associated with engineered counterparts.

Addressing global issues like water desalination and anti-icing involves adopting scalable, cost-effective methods, such as sol–gel and chemical vapor deposition, and innovative low-energy solutions like plasma cannon deposition. While advanced techniques like atomic layer deposition offer precision, methods like dip coating, spin coating, and printing provide efficient, cost-effective alternatives for large-scale production. Sustainability considerations highlight the importance of affordability and reduced waste in nanomaterial fabrication, driving the responsible evolution of nanotechnology. The diverse applications of nanomaterial deposition, including sensing, anti-icing, antibacterial surfaces, and filters, underscore the versatile potential of tailored nanomaterials in enhancing specific properties. Ensuring high precursor purity is critical, as impurities can negatively affect nanomaterial quality and performance, emphasizing the need to maintain purity for desired characteristics.

## Figures and Tables

**Table 1:** Deposition of nanomaterials on ceramic substrates.

Application	Technique	Nanomaterial	Matrix	Year	Reference
Effect on structural and optical properties (With increasing film thickness, the dielectric properties, absorption coefficient, and band gap energy have increased)	Vertical deposition angle and high vacuum using electron gun evaporation method	Tantalum oxidenanolayers	Glass substrate	2021	[25]
Low-cost methodology	Spray deposition	Graphene nanoplatelets	Carbon fiber–reinforced polymer	2020	[26]
Low-cost and easy way for the preparation of 1D ZnO nanostructures	Electrochemical deposition	ZnO nanorod arrays	ITO glass substrates	2015	[27]
Antibacterial properties	Tollen’s reaction	Silver nanoparticles	Inorganic and organic substrate surfaces (Metallic, polymeric, ceramic)	2004	[28]
Sensing and catalysis	Electroless deposition	Au nanoparticles	Different substrates	2020	[29]
Modify physical properties	Physical vapor deposition	Titanium nanolayers	Glass substrates	2013	[36]
Low cost to provide reliable bond strength	In situ deposition	Nano-Silica	Yttria–Tetragonal Zirconia Polycrystal (Y–TZP)	2015	[37]

**Table 2:** Deposition of nanomaterials on polymeric substrates.

Application	Technique	Nanomaterial	Matrix	Year	Reference
Improve mechanical properties and mechanical erosion by gases and particulate erosion	Chemical vapor deposition	Hydrocarbon gases	Nanoporous structure (Polymer composite)	2022	[39]
Enhance bond properties	Electrophoretic deposition	Nano-Silica	Carbon Fiber surfaces	2020	[40]
Antimicrobial properties	In situ deposition	Nano-Cu_2_O	Electrospun chitosan nanofibrous	2021	[41]
Photocatalytic activation properties	Sol–gel method	NiO nanoparticles	Cotton	2021	[42]
Wound healing	Pulsed laser deposition	Ag NPs	Selenium/carbonated hydroxyapatite/e polycaprolactone nanofibrous	2020	[43]
Rapid and cost-effective method for deposition	Microblast deposition technique	Nanostructured TiO_2_	Polymer, conductive glass, and metallic substrate	2013	[44]
Electrocatalytic activities and improve tolerance of CO	In situ electro-deposition	Pt micro–nano clusters	{[PMo_12_O_40_]^3–^/PAMAM}	2012	[45]
Electrocatalyst	Electrochemical deposition	Au–Pt	PAP films	2023	[46]

**Table 3:** Deposition of nanomaterials on metallic substrates.

Application	Technique	Nanomaterial	Matrix	Year	Reference
Improve mechanical properties	Electrostatic layer-by-layer	Diamond Nanoparticles (SiO_2_, TiO_2_)	Si substrate	2022	[54]
Improve optical and electrical properties	Plasma-Assisted Hot-Filament Evaporation	Silver nanoparticles	Indium Tin Oxide (Metallic)	2020	[55]
Improve mechanical properties	Direct Laser Deposition	Nano-TiB whiskers	Ti–6Al–4 V	2022	[49]
NR	Electrochemical deposition	Si nanospheres	Au and graphene Coated Cu	2022	[56]
High reproducibility (Industrial applications)	Chemical vapor deposition	Carbon nano-onions	Fe/Fe_3_C	2022	[57]
Biocompatibility	Atomic Layer deposition	Nano Sn–O_2_	Titanium	2022	[53]
Influence on microstructure (Decrease of SiCp size—The reaction between SiC and Aluminum matrix was more intense)	Laser metal deposition	Nano-SiCp	AlSi_10_Mg Alloy	2022	[58]
Homogeneously equiaxed grains and no cracks compared	Direct laser deposition	TiAl (Metal matrix composite)	Ti—55Al—7.5 Nb/Ti—55	2022	[59]
Improve biological and physical characteristics	Electrophoretic deposition	Chitosan-reinforced Baghdadite ceramic nanoparticles	Stainless steel 316 L	2022	[60]
Corrosion protection	Electrophoretic deposition	Polyaniline (PANI) Nanofibers	Copper	2022	[61]
High active hydrogen evolution reaction	In situ deposition	Pt nanoparticles	Nano-pore stainless steel	2021	[62]
Corrosion Protection	Electrochemical deposition under ultrasonic condition	CaP/SiO_2_ (Nanosized silica)	Titanium	2021	[63]
Enhanced microhardness properties	Electroless deposition	SiC and Ni–P	Aluminum alloy lm25	2021	[64]
Electro-analytical application	Plasma-enhanced chemical vapor deposition	Large-area nanocrystalline graphite film (NCG)	SiO_2_	2019	[34]
Improve the microhardness, microstructure, and wear rate	Electroless deposition	SiC nanocoating	Aluminum alloy	2021	[65]
Improve etching resistivity	Chemical vapor deposition (CVD)	Amorphous carbon films	Nanocrystalline graphite	2021	[66]
Improve mechanical and corrosion properties	Machining process and heat treatment	Al_2_O_3_ Nanoparticles	Steel	2018	[67]
Improve mechanical properties	Laser metal deposition	Cr nanoscale precipitates	Cu alloy	2021	[68]
Improve mechanical properties and biocompatibility	Electrophoretic deposition	Nano HA–SiC	AISI 316l medical grade stainless	2021	[69]
Hemocompatibility and MG–63 cell proliferation	Electrophoretic deposition	Bioactive glass (BG) and reduced graphene oxide (rGO)—bioglass nanohybrid (G–BG)	Anodized TiO_2_ nanotubes	2020	[70]
Improve mechanical properties (Hardness)	Cold spray process	MgO Nanoparticle	Steel	2018	[71]
Aerogel for high-performance asymmetrical supercapacitor	Electrophoretic deposition	Carbon nanorod/reduced graphene oxide	Nickel foam	2020	[72]
New nanostructures with specifically a new property	RF sputtering using dynamic glancing angle deposition	Nanostructured CrN	Crystalline silicon	2019	[73]
Efficient removal of organic pollutants	Atomic layer deposition	TiO_2_ nanocrystal	Activated carbon	2021	[74]
Optoelectronic applications	Atomic layer deposition	ZnSe nanowires	Polycrystalline CdS	2019	[75]
Improve corrosion resistance	electroplating	SnO_2_ nanoparticles	Zn composite	2021	[76]
Suitable for nanodevice applications	Metalorganic chemical vapor deposition	GaN nanowires	Nano-patterned Si (111)	2011	[77]
Improve mechanical properties (Hardness and Young’s modulus)	Chemical vapor deposition (CVD)	TiCN	Si substrate	2020	[78]
Enhanced surface roughness for superhydrophobicity	Electrochemical deposition	Nano-hierarchical structure	Copper film	2016	[79]
Polymer/inorganic hybrid solar cells	Pulsed laser deposition	ZnSe nanoneedles	Silicon substrates	2013	[80]
Antioxidative properties and excellent thermal stability up to 500 °C in air	Electrophoretic deposition	Polyimide nanocoating	Carbon fibers	2011	[81]
Semiconductors with unique properties	Atomic Layer Deposition	Tungsten oxide nanocrystals	Si substrates	2017	[82]
Improve mechanical properties (Hardness and wear resistance) and the resistance to erosion—enhanced corrosion	Electrolytic deposition	Ni–Co–SiC nanocoating	Carbon Steel	2011	[83]
Mechanism of self-organized formation or nano multilayer	Sputtering deposition	Nano-multilayer structure	Carbon-thin copper film	2017	[84]
Less defect density	Pulsed laser deposition	Nano-zinc ferrite	Carbon Steel	2016	[85]
Photoluminescence properties	Electroless deposition	Cu_2_O nanocrystallites	Silicon nanowire arrays	2013	[86]
Emit into ultraviolet wavelengths and insert it into optoelectronic devices.	Chemical vapor deposition	Aluminum nitride nanodots	Si (111)	2011	[87]
Aerospace application (The mechanical properties such as hardness and wear resistance are improved by heat treatment)	Electroless deposition	Nano Ni–B–Sn	7075—T6 aluminum alloy	2019	[88]
Biocompatibility for implants in biological materials	Electrochemical deposition and chemical treatment	Nano-hydroxyapatite	Carbon fibers	2016	[89]
Improve corrosion resistance and surface topography	Electrostatic spray deposition	Nano-ceramic	Orthopedic Implant (Metallic)	2015	[90]
Improve the corrosion resistance, pitting potential, and more compact material	Combined physical vapor deposition and electrochemical deposition	Nano-TiO_2_/FHA	Mg–Zn–Ce Alloy	2014	[91]
An alternative method to place coating on steel	Active screen plasma nitriding	Nanosized titanium nitride	Steels	2011	[92]
Oil–water separation with efficiency up to 95.8% ± 0.9%	Electrophoretic deposition	Nano-aluminum	Stainless steel	2018	[93]

**Table 4:** Summary of driving forces for the deposition and transformation of nanoparticles.

Technique	Driving Force	Reference
Deposition by electron gun evaporation	Electric Fields, Thermal Effects	[25,30]
Physical Vapor Deposition (PVD)	Surface Interaction Forces, Thermal Effects	[31,32,33]
Plasma-Enhanced Chemical Vapor Deposition (PECVD)	Chemical Affinity, Electric Fields	[34,35]
Simultaneous and consecutive electrochemical deposition	Electrochemical Deposition, Kinetic Factors	[46]
Electrophoretic Deposition	Electric Fields, Surface Interaction Forces	[40]
Laser Metal Deposition	Thermal Effects, Kinetic Factors	[48]
Electrochemical Deposition	Electrochemical Deposition, Kinetic Factors	[46]
Atomic Layer Deposition (ALD)	Chemical Affinity, Electric Fields	[51,52,53]
Thermophoretic deposition	Thermal Effects, Fluid Dynamics	[94,95,96,97]
Supercritical deposition	Fluid Dynamics, Thermal Effects	[98]
Spin Coating	Fluid Dynamics, Kinetic Factors	[99,100,101,102]
Dip Coating	Fluid Dynamics, Kinetic Factors	[103,104,105,106]

**Table 5:** Required purities in precursors employed in deposition techniques.

Technique	Precursor Purity	Nanomaterial	Year	Reference
Vertical Deposition	99.90%	Tantalum oxide nanocoatings	2021	[25]
Plasma-Enhanced Deposition	99.90%	Silver nitride nanoparticles	2004	[28]
Pulsed Laser Deposition	99.90%	Silver nanoparticles	2020	[43]
Physical Vapor Deposition	98.00%	Titanium nanocoatings	2013	[36]
Electrochemical Deposition	99.99%	Gold nanoparticles	2023	[46]
Electrophoretic Deposition	99.00%	Copper nanoparticles	2021	[135]
Electrophoretic Deposition	99.00%	Gold nanocoatings	2020	[136]
Electroless Deposition	99.90%	Gold nanoparticles	2019	[137]
Atomic Layer Deposition	97.00%	Palladium nanocoatings	2015	[138]

## Data Availability

Not applicable.
